# RNAi-Mediated Silencing of the Chitinase 5 Gene for Fall Webworm (*Hyphantria cunea*) Can Inhibit Larval Molting Depending on the Timing of dsRNA Injection

**DOI:** 10.3390/insects12050406

**Published:** 2021-04-30

**Authors:** Xun Zhang, Yue Wang, Sufang Zhang, Xiangbo Kong, Fu Liu, Zhen Zhang

**Affiliations:** Key Laboratory of Forest Protection of National Forestry and Grassland Administration, Research Institute of Forest Ecology, Environment and Protection, Chinese Academy of Forestry, Beijing 100091, China; zhx3355@126.com (X.Z.); cafwy1016@163.com (Y.W.); Zhangsf@caf.ac.cn (S.Z.); xbkong@sina.com (X.K.); liufu@sina.com (F.L.)

**Keywords:** *Hyphantria cunea*, chitinase, molting, RNAi efficiency, transcriptome analysis

## Abstract

**Simple Summary:**

The fall webworm, *Hyphantria cunea*, is a worldwide invasive pest causing serious ecological and economic damage. The use of RNAi is a feasible strategy for controlling this pest. In this study, we evaluated the importance of the chitinase 5 gene (*HcCht5*) in the development of *H. cunea* larvae. We found that the RNAi-mediated silencing of *HcCht5* arrested molting and caused larval mortality depending on the dsRNA injection time. The silencing of *HcCht5* down-regulated genes that were related to chitin metabolism, molting hormone signaling, and detoxification metabolism. Our findings indicate that *HcCht5* is an important gene in regulating larval development and a promising target for RNAi-mediated pest management of the pest *H.* *cunea*.

**Abstract:**

Chitinases, which are crucial enzymes required for chitin degradation and reconstruction, are often selectively considered to be effective molecular targets for pest control due to their critical roles in insect development. Although the *Hyphantria cunea* chitinase gene has been reported previously, its sequence characteristics, gene function, and feasibility as a potential target for pest management were absent. In the present study, we characterized the *H. cunea* chitinase gene and designated it *HcCht5*. Phylogenic and domain structure analysis suggested that *HcCht5* contained the typical chitinase features and was clustered into chitinase group I. Tissue-specific and developmental expression pattern analysis with Real-Time Quantitative PCR (RT-qPCR) showed that *HcCht5* was mainly expressed in the integument tissues and that the transcript levels peaked during molting. RNA interference (RNAi)-mediated silencing of *HcCht5* caused 33.3% (2 ug) and 66.7% (4 ug) mortality rates after double-stranded RNA (dsRNA) injection. Importantly, the interference efficiency of *HcCht5* depended on the injection time of double-stranded RNA (dsRNA), as the pre-molting treatment achieved molt arrest more effectively. In addition, transcriptome sequencing (RNA-seq) analysis of RNAi samples demonstrated silencing of the down-regulated *HcCht5* genes related to chitin metabolism and molting hormone signaling, as well as genes related to detoxification metabolism. Our results indicate the essential role of *HcCht5* in *H. cunea* development and detail the involvement of its gene function in the larval molting process.

## 1. Introduction

Chitin, a linear polymer made of *N*-acetyl-β-D-glucosamine (GlcNAc) linked by β-1,4 glycosidic bonds, is an important component of the epidermis and peritrophic matrix in insects [1]. The balance of the chitin content is particularly essential for insect development (molting) and metamorphosis. Chitin synthesis and degradation occur simultaneously during insect molting, when the old cuticle is degraded and replaced with the new cuticle formed by the underlying epidermal cells [2]. Multiple enzymes are involved in this process of chitin metabolism. Chitinases (*Chts*, E.C.3.2.1.14), which belong to glycoside hydrolase family 18 (GH18), are key enzymes in the degradation of chitin. The function of chitinase is to hydrolyze the linear polymer of chitin and degrade chitin into low-molecular-weight chitooligosaccharides. Insect chitinases are essential for insect survival and development. They appear to play roles in cuticle turnover, wing expansion, digestion, immunity and natural defense [1,3]. Insect chitinase was first cloned from *Manduca sexta* [4]. Since then, hundreds of chitinase genes have been continuously reported from different insect species, such as *Tribolium castaneum* [3], *Spodoptera litura* [5], *Choristoneura fumiferana* [6], *Helicoverpa armigera* [7], *Spodoptera exigua* [8], *Locusta migratoria* [9], and *Sogatella furcifera* [10].

Currently, chitinases and chitinase-like proteins are classified into 11 groups (groups I to X, and the Lepidoptera-specific group h) based on their sequence similarities, domain architectures, tissue-specificity of expression, and functions [11,12,13]. Among them, group I (*Cht5*) has been relatively well characterized. The transcripts of *Cht5s* are mainly detected in the epidermis and the gut [7], which suggests that *Cht5s* may be involved in chitin turnover in the cuticular exoskeleton and peritrophic membrane. Heterologous expression of *Cht5* of *Drosophila melanogaster* and *T. castaneum* has also been successfully performed in the Hi-5 cell lines, and the recombinant protein showed high levels of chitinolytic activity [13]. At present, most studies on *Cht5* revealed its function in pupal–adult or nymph–adult molting in coleopteran, orthopteran, and homopteran insects using RNA interference (RNAi) technology. For instance, in *T. castaneum*, *TcCht5* was down-regulated by the injection of *TcCht5*-specific double-stranded RNA (dsRNA) into larvae, which led to pupal–adult molting arrest [3]. In *L. migratoria*, two *Cht5* genes, *LcCht5-1* and *LcCht5-2*, were identified. The RNAi of *LcCht5-1* disrupted molting from nymphs to adults [14]. In *Sogatella furcifera*, silencing of *SfCht5* also affected the nymph–adult transition [10]. However, the function of *Cht5* in larvae-larvae molting in Lepidoptera has been rarely studied.

The fall webworm, *Hyphantria cunea* Drury, is a Lepidoptera insect belonging to the family of Arctiidae. *H. cunea* is a worldwide invasive pest, and the larval stage is the main stage in which the larvae can attack and feed on more than 600 host plants, causing serious ecological and economic damage [15]. Currently, monitoring and control of *H. cunea* is primarily achieved through chemical insecticides, which are environmentally unfriendly. Some parasitoid wasps, sex pheromone traps, and biopesticides are also used, but are inefficient and costly [16,17,18,19]. The identification of novel management strategies is urgently required to control this pest. In recent years, RNAi-based pest management has been increasingly studied as a novel insect control strategy and the publication of genome and transcriptome sequencing for *H. cunea* also provided a molecular basis for *H. cunea* control [20,21,22]. However, potentially effective genetic targets and innovative strategies to control this invasive pest are still lacking. Chitinase 5 genes from different insects have been identified as potential silencing targets and resulted in abnormal molting and increased mortality in the pupal–adult or nymph–adult process [23,24]. However, for *H. cunea*, the larval stage is the important damage stage and the key period for its control. Little research has focused on chitinase genes from *H. cunea*. Although a chitinase gene (*Cht5*, Accession number: U86877) was cloned by Kim et al. in *H. cunea* in 1998 [25], its sequence characteristics, gene function, and feasibility as a potential target for pest control were absent. To research the function of this chitinase gene in the *H. cunea* larvae molting process, we further identified and analyzed the function of this gene using the RNAi method and considered the possibility of its use as one of the competitive molecular targets for *H. cunea* control.

In the present study, we identified and analyzed the domain structure of *H. cunea* chitinase gene (*HcCht5*), a group I gene, and profiled its tissue-specific and developmental expression patterns. RNAi and transcriptome sequencing (RNA-seq) were used to gain insights into the biological function of *HcCht5*. Our results revealed the crucially important role of *HcCht5* during larval molting and in the promotion of the development of RNAi-based management of *H. cunea* control.

## 2. Materials and Methods

### 2.1. Insect Rearing

*H. cunea* larvae were kindly provided by the laboratory of Insect Virus Research Center, Chinese Academy of Forestry. They were reared on an artificial diet under a 14 h light: 10 h dark photoperiod at 26 (±1) °C with 75% (±10%) relative humidity.

### 2.2. Cloning and Sequencing of the HcCht5 cDNA

The total RNA was isolated from the third instar larvae on the second day after molting using the Trizol Plus reagent (Ambion, Austin, TX, USA), following the manufacturer’s recommended protocol. The RNA concentration and quality were assessed using a spectrophotometer (Denovix, Wilmington, DE, USA) and 1% agarose gel electrophoresis. cDNA synthesis was performed by the GoScript™ Reverse Transcription System kit (Promega, Madison, WI, USA) with an oligo (dT)_15_ primer, and 1 ug of total RNA was used per reaction.

The full-length coding sequence of *HcCht5* was amplified using PrimeSTAR^®^ Max DNA Polymerase (Takara, Shanghai, China). PCR primers were designed based on the reported *H. cunea* chitinase gene (Accession number: U86877) with the Primer Premier 5 software. The primer sequences are listed in Table S1. The PCR program used was as follows: 94 °C for 3 min; 35 cycles of 94 °C for 30 s, 55 °C for 30 s and 72 °C for 2 min; and 72 °C for 10 min. The PCR products were purified and cloned into a pEASY-Blunt3 vector (TransGen, Beijing, China) and sequenced at Sangon Biotech (Co., Ltd., Beijing, China).

### 2.3. Deduced Amino Acid Sequence Analysis of HcCht5

The amino acid sequences were deduced using the Translate tool on the ExPASy Proteomics website (http://web.expasy.org/translate/) (accessed on 20 February 2020). The molecular weight and isoelectric point (*pI*) of HcCht5 were predicted using the Compute pI/Mw tool (https://web.expasy.org/compute_pi/) (accessed on 20 February 2020). SMART domain analysis (http://smart.embl-heidelberg.de/) (accessed on 20 February 2020) and SignalP 4.1 Server (http://www.cbs.dtu.dk/services/SignalP/) (accessed on 20 February 2020) were used to predict the domain architecture and signal peptide. Percent identity analysis of HcCht5 was conducted by NCBI blastp (https://blast.ncbi.nlm.nih.gov/Blast.cgi) (accessed on 25 February 2020). To compare the amino acid sequences and catalytic domains of HcCht5 along with other insect orders, multiple amino acid sequence alignment was carried out using Clustal X software [26], and the identities among Chts were analyzed by GeneDoc software (http://wwwpscedu/biomed/genedoc) (accessed on 25 February 2020). Four conserved motifs were identified based on the references previously described by Arakane and Muthukrishnan [1] and Zhang et al. [27]. The known insect *Cht5* genes in other insect orders deposited in GenBank were used to construct phylogenetic trees using the MEGA 6.0 software package with the neighbor-joining method. Bootstrap analysis was performed, and the robustness of each cluster was confirmed in 1000 replications.

### 2.4. Tissue-Specific and Developmental Expression Analysis of HcCht5

For tissue-specific expression analysis, different tissues (head, integument, gut, and fat bodies) were dissected from larvae on the second day of the fifth instar stadium (L5D2). The larvae were first kept on ice for 3 min and then dissected with sterile insect scalpels under a zoom stereomicroscope (Olympus, SZX7). The heads from ten larvae and other tissues (integument, gut, and fat bodies) from three larvae were pooled as one treatment. Each treatment contained three biological replicates. For developmental expression analysis, the whole larva was sampled and used for developmental expression analysis. The first to fifth instar larvae (L1 to L5) larvae on the third day of the stadium (D3) and the fourth instar larvae from day 1 to day 5 of the stadium (L4D1 to L4D5) were collected. Samples from each developmental stage contained three biological replicates. At least 100 mg of tissue or larvae were sampled per replicate. All collected samples were immediately frozen in liquid nitrogen and stored at −80 °C.

### 2.5. RT-qPCR

To screen for the stable reference genes of *H. cunea*, qRT-PCR was employed to analyze the expression of four candidate reference genes (*β-actin*, *β-tubulin*, *GAPDH*, and *EF1α*) at different developmental stages (L1 to L5) and different tissues (head, integument, gut, and fat bodies), respectively. RT-qPCR was performed using the SuperReal PreMix Plus (SYBR Green) kit (TIAN GEN, Co., Ltd., Beijing, China) with a 20 μL reaction. Each reaction contained the following: 10 μL 2 × SuperReal PreMix Plus (SYBR Green) solution, 0.6 μL forward and reverse primers in a final concentration of 10 μM, 7.8 μL nuclease free water, and 1 μL of undiluted cDNA. RT-qPCR was carried out using a LightCycler 480 II (Roche, Basel, Switzerland) with the following conditions: 95 °C for 3 min, followed by 45 cycles at 95 °C for 5 s, and 60 °C for 30 s. Each reaction included three technical repetitions. According to the analysis by NormFinder (https://www.moma.dk/normfinder-software/) (accessed on 15 March 2020), the stabilities of the four candidate reference genes were analyzed.

All RT-qPCR experiments were conducted with the methods above. Each treatment included three biological replicates and three technical repetitions. The relative mRNA levels of the target genes were calculated using the 2^−∆∆Ct^ method by normalizing them to the expression of the screened reference gene. All the PCR primers were designed using the Primer Premier 5 software. The primer sequences are listed in Supplementary Table S1. Melting curve analyses were performed for all the primers.

### 2.6. dsRNA Synthesis and RNAi of HcCht5

The dsRNA was synthesized using a T7 RiboMAX™ Express RNAi System (Promega, Madison, WI, USA) in accordance with the manufacturer’s instructions. T7 promoter sequences were tailed to the 5′-ends of the DNA templates by PCR amplification. The primer sequences are listed in Supplementary Table S1. Template DNA and single-stranded RNA were removed from the transcription reaction by DNase and RNase treatments, respectively.

For RNAi, larvae from the last day of the third stadium (L3D5) with similar sizes and growth conditions were selected, 2 ug and 4 ug of ds*HcCht5* or ds*GFP* (control) solution were injected into each larva through the abdominal side between the fourth and fifth abdominal segments, respectively, using a microinjector (Hamilton, Bonaduz, Switzerland). A total of 12 larvae were treated for each group, three larvae (replicates) were randomly sampled from each treatment group at 12 h and 24 h post treatment, immediately frozen in liquid nitrogen, and stored at −80 °C. RNA extraction and RT-qPCR detection of the relative transcript level of *HcCht5* were conducted as described above. For RNAi phenotype observation, another 30 larvae were treated for each group, the abnormal rate was investigated at the molting stage, and the mortality rate was investigated five days post treatment.

To research the RNAi efficiency of *HcCht5* at different injection times, 30 larvae were separately injected with 4 ug ds*HcCht5* from day 1 to day 5 of the fourth instar larvae stadium (L4D1 to L4D5). The control group was injected with the same amount of ds*GFP*. The abnormal rate and mortality rate were investigated at the molting stage and five days post molting, respectively.

### 2.7. RNA-Seq and Analysis

At the first day of the fourth stadium, the larvae were injected with 2 ug ds*HcCht5* or ds*GFP* (control). Three larvae (biological replicates) from each treatment were collected 12 h post injection, immediately frozen in liquid nitrogen, and then stored at −80 °C. For mRNA sequencing, the total RNA was extracted as described above. RNA degradation and contamination were monitored on 1% agarose gels. RNA purity was checked using the NanoPhotometer^®^ spectrophotometer (IMPLEN, Westlake Village, CA, USA). The RNA concentration was measured using a Qubit^®^ RNA Assay Kit in Qubit^®^ 2.0 Flurometer (Life Technologies, Carlsbad, CA, USA). The RNA integrity was assessed using the RNA Nano 6000 Assay Kit of the Agilent Bioanalyzer 2100 system (Agilent Technologies, Santa Clara, CA, USA).

A total amount of 3 μg RNA per sample was used as input material for the RNA sample preparations. Sequencing libraries were generated using NEBNext^®^Ultra™ RNA Library Prep Kit for Illumina^®^ (NEB, Ipswich MA, USA) following the manufacturer’s recommendations, and index codes were added to attribute sequences to each sample. The library preparations were sequenced on an Illumina HiSeq2000 platform (Illumina, San Diego, CA, USA) by Biomarker Technologies (Co., Ltd., Beijing, China).

The raw data outputs from the Illumina equipment were trimmed for adapters, and polyA/T tails and low-quality reads (Q20 less than 20) were removed to obtain high-quality clean reads. The clean reads were assembled to produce unigenes using Trinity [28]. The assembled unigenes were aligned with the NR databases using BLAST (http://blast.ncbi.nlm.nih.gov/Blast.cgi) (accessed on 10 August 2020) with a cut-off E-value of 10^−5^.

The unigene abundance was measured as the fragments per kilobase of transcript per million mapped reads (FPKM) using RSEM [29]. The identification and counting of differentially expressed genes (DEGs) between ds*GFP*- and ds*HcCht5*-treated samples were conducted with the DESeq R package (1.10.1) at a False Discovery Rate (FDR) of ≤0.05 and a log_2_ fold change of ≥1.5, followed by hierarchical clustering based on the expression values. DEGs were validated by RT-qPCR as described above. DEG analysis was performed using BMKCloud (www.biocloud.net) (accessed on 10 September 2020).

### 2.8. Statistical Analysis

For the analysis of *HcCht5* expression patterns in different tissues and at different developmental stages, one-way analysis of variance followed by Tukey’s test was applied. The other data were analyzed statistically using an independent sample Student’s *t*-test. In the figures, different letters above the bars represent significant differences in the *HcCht5* expression between the samples (*p* < 0.05), while asterisks are used to indicate significant differences (*, *p* < 0.05).

## 3. Results

### 3.1. Sequence Analysis of HcCht5

**Figure 1:**
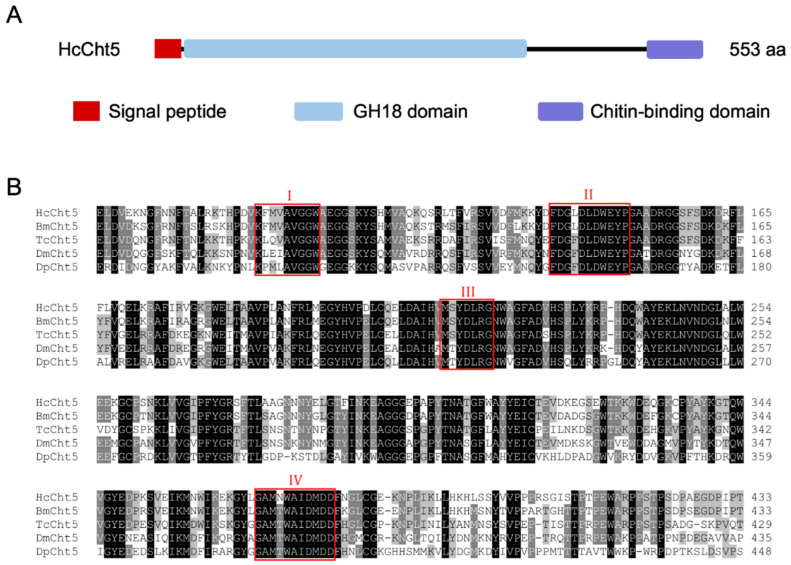
Domain architectures and catalytic motif analysis of *H. cunea* chitinase (HcCht5). (**A**) Domain architectures of HcCht5. (**B**) Conserved domains in the glycoside hydrolase family 18 (GH18) of HcCht5 and four other group I chitinases (BmCht5, TcCht5, DmCht5, and DpCht5). The conserved and similar amino acid residues are labeled with black (100% identity) and gray (60–80% identity) backgrounds, respectively. Red-boxed regions are the four conserved motifs (I–IV) represented by the sequences KXXXAVGGW, FDGXDLDWEYP, MXYDLRG, and GAMXWAIDMDD, where X is a nonspecific amino acid. Four insect chitinases belonging to chitinase group I (BmCht5, TcCht5, DmCht5, and DpCht5) were used for catalytic motif analysis. Their GenBank accession numbers are listed in Table S2.

The full-length coding sequence of the *HcCht5* gene had a 1662 bp ORF encoding 553 amino acid residues with a predicted molecular weight of 61.97 kDa and a predicted *pI* of 4.96. The deduced amino acid sequence of HcCht5 showed 80.07% identity with BmCht5. The domain architecture of HcCht5 contained a signal peptide (amino acids 1–20), a catalytic domain (GH18 domain, amino acids 24–376), and a chitin-binding domain (amino acids 496–553) (Figure 1A). Catalytic motif analysis showed that HcCht5 possesses four conserved catalytic domains: KXXXAVGGW, FDGXDLDWEYP, MXYDLRG, and GAMXWAIDMDD, where X is a nonspecific amino acid (Figure 1B). These motifs are considered the catalytic active sites of chitinase. Phylogenetic analysis showed that HcCht5 exhibited a high homology with BmCht5 and was clustered in the branch of group I chitinases (Figure S1).

### 3.2. Tissue-Specific and Developmental Expression of HcCht5

**Figure 2:**
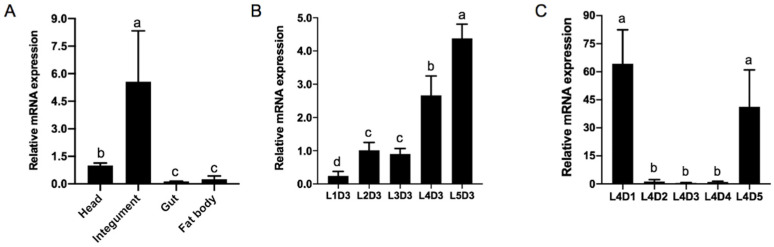
The expression of *HcCht5* in *H. cunea* by RT-qPCR. (**A**) The expression of *HcCht5* in different tissues of day 3 of the fifth instar larvae. (**B**) The expression of *HcCht5* on the third day of each larval stadium (L1D3–L5D3). (**C**) The expression of *HcCht5* on each day of the fourth larval stadium (L4D1–L4D5). Error bars represent the standard error of the calculated means based on three biological replicates. Different letters above the bars represent significant differences in the *HcCht5* expression between the samples (one-way analysis of variance followed by Tukey’s test, *p* < 0.05).

RT-qPCR was used to analyze the spatiotemporal expression levels of *HcCht5*. To screen for stable reference genes of *H. cunea*, four candidate reference genes (*β-actin*, *β-tubulin*, *GAPDH*, and *EF1α*) were evaluated for their expression stability in different developmental stages and different tissues. Finally, *β-Actin* showed the most stability and was used as the reference gene for RT-qPCR analysis (Table S3). To investigate the tissue-specific expression of *HcCht5*, different tissues (head, integument, gut, and fat bodies) from the fifth instar larvae were dissected and tested. The results showed that *HcCht5* was expressed in all the tested tissues and most highly expressed in the integument (Figure 2A).

To investigate the developmental expression of *HcCht5*, we detected the expression level of *HcCht5* in day 3 larvae from the first instar to the fifth instar (L1D3–L5D3) and in larvae from the first day to the last day of the fourth instar (L4D1–L4D5), respectively. The results showed that *HcCht5* was expressed at a low level in the early stage (L1 to L3) and a high level at the fourth and the fifth instar (L4 and L5) (Figure 2B). The *HcCht5* mRNA displayed an extremely high expression level at L4D1 and L4D5, but an extremely low expression at L4D2 to L4D4 (Figure 2C). This indicates that *HcCht5* was significantly up regulated during molting and rapidly disappeared during inter-molting, implying a conceivable role of *HcCht5* during the molting process.

### 3.3. RNAi of HcCht5

**Figure 3:**
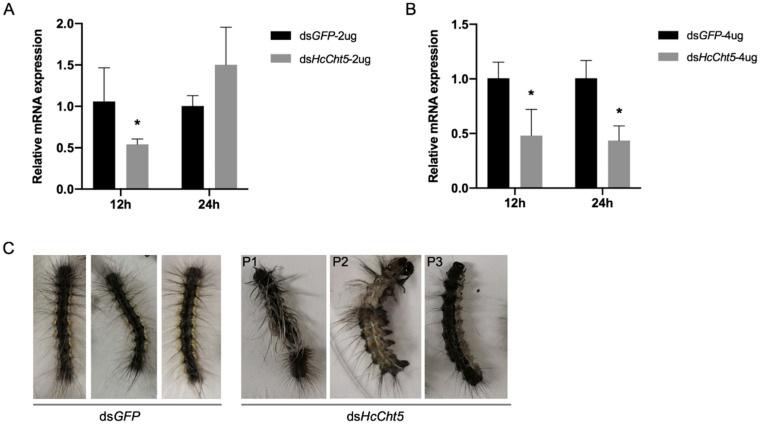
RNAi efficiency of *HcCht5.* (**A**) The relative mRNA expression levels of *HcCht5* after injection with 2 ug ds*HcCht5* and ds*GFP*. (**B**) The relative mRNA expression levels of *HcCht5* after injection with 4 ug ds*HcCht5* and ds*GFP*. dsRNAs were injected in L3D5 instar (the day before molting) larvae. The relative mRNA expression levels of *HcCht5* were determined using RT-qPCR at 12 h and 24 h post injections. Error bars represent the standard error of the calculated means based on three biological replicates. Asterisks indicate significant differences (Student’s *t*-test, *p* < 0.05). (**C**) The abnormal molting phenotypes of *H. cunea* larvae after injection with ds*HcCht5*. Insects injected with ds*GFP* were able to molt successfully, while the ds*HcCht5*-injected insects displayed three abnormal molting phenotypes: P1, some larvae could shed the epidermis but did not molt completely; P2, some larvae stopped molting halfway; and P3, some larvae completely failed to molt.

To investigate the effect of *HcCht5* on *H. cunea* molting, RNAi was performed by the injection of ds*HcCht5* and ds*GFP* (control). Since *HcCht5* was highly expressed pre-molting, we selected larvae on the fifth day of the third instar (the day before molting) for RNAi. RT-qPCR was carried out to test the transcription level of *HcCht5* after treatment. The results showed that the expression of *HcCht5* was effectively reduced at 12 h but recovered at 24 h after the injection of 2 ug ds*HcCht5* against ds*GFP* (Figure 3A). In contrast, a concentration of 4 ug ds*HcCht5* resulted in significant silencing at both 12 h and 24 h post injection (Figure 3B). In addition, phenotypic observations suggested that all the larvae in the control group could molt normally, whereas the ds*HcCht5* injection group showed 33.3% (2 ug) and 66.7% (4 ug) abnormal molting (Table 1). These abnormalities were characterized by the fact that some larvae could shed the epidermis but did not molt completely, some larvae stopped molting halfway, and some could not molt at all (Figure 3C). Finally, the ds*HcCht5* injection resulted in 33.3% (2 ug) and 66.7% (4 ug) mortality rates at five days post treatment, while no death was observed in the control group (Table 1). These results demonstrated the crucially important role of *HcCht5* during *H. cunea* larval molting.

Considering the expression pattern of *HcCht5*, which displayed an extremely high expression during ecdysis and then followed a sharp decline after ecdysis (Figure 2C), we wondered whether the injection time could affect the RNAi efficiency. Therefore, injections of dsRNA at different stages of the fourth instar larvae (L4D1–L4D5) were conducted. We found that only the treatment at L4D4 and L4D5 resulted in the molting defect and lethal phenotype, whereas no phenotypic changes were detected for treatment at L4D1, L4D2 and L4D3 (Table 2). In addition, compared with the ds*GFP* control, injection of ds*HcCht5* at L4D5 resulted in a higher abnormal rate (26.7%) and mortality rate (26.7%) than the injection at L4D4, which showed a 10% abnormal rate and 10% mortality rate, respectively (Table 2). These results indicated that the injection time of ds*HcCht5* could affect the RNAi efficiency of *HcCht5*.

### 3.4. Differentially Expressed Gene (DEG) Analysis after HcCht5 RNAi

**Figure 4:**
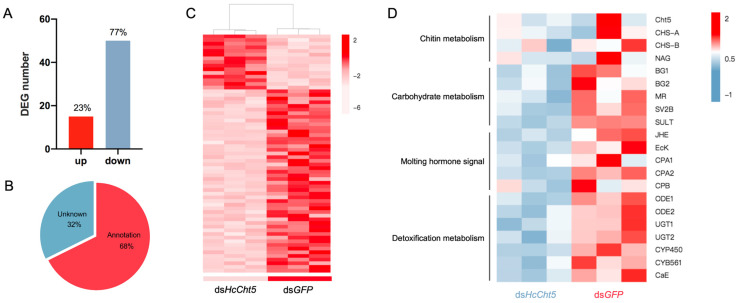
Differentially expressed gene (DEG) analysis of the ds*HcCht5*- and ds*GFP*-treated groups. (**A**) The number of DEGs in the ds*HcCht5*-treated group compared with the ds*GFP* group. Up indicates up regulated DEGs, and down indicates down-regulated DEGs. (**B**) The proportion of annotation and unknown DEGs. (**C**) Expression profiles of all DEGs between the ds*HcCht5* and ds*GFP* groups. Each group contained three repeated samples. The color scale indicates the different expression levels, which were normalized as log_10_ fragments per kilobase of transcript per million mapped reads (FPKM) values. (**D**) Expression profiles of some of the most down-regulated DEGs and other interesting DEGs in the ds*HcCht5* and ds*GFP* groups. Each group contained three repeated samples. The color scale indicates the different expression levels. The letters on the right indicate putative gene names in *H. cunea*. Abbreviations: Cht5, chitinase 5; CHS-A and CHS-B, chitin synthase A and B; NAG, β-*N*-acetylglucosaminidase; BG1 and 2, β-glucosidase precursor 1 and 2; MR, putative mandelate racemase; SY2B, synaptic vesicle glycoprotein 2B-like; SULT, sulfotransferase; JHE, juvenile hormone esterase; EcK, ecdysteroid kinase; CPA and B, carboxypeptidase A and B; ODE1 and 2, odorant-degrading enzymes; UGT 1 and 2, UDP-glycosyltransferases; CYP450, cytochrome P450; CYB561, cytochrome b-561 domain containing protein; and CaE, carboxylesterase.

To investigate the effect of *HcCht5* RNAi on other genes, RNA-seq analysis was used to identify genes that were differentially expressed after *HcCht5* RNAi. A total of 154,927,972 clean reads were yielded after filtering out the adapter sequences and low-quality reads (Table S4). The gene expression profile of biological replicates from the same group showed a high correlation coefficient (Figure S2). According to the results of the transcriptome assembly, 54,852 unigenes were obtained and annotated to the NR database. Compared with ds*GFP*, 65 unigenes were identified as being differentially expressed in ds*HcCht5* (log2FC > 1.5, FDR < 0.05), among which 77% were down-regulated and 23% were up regulated (Figure 4A, Table S5). The heat map shows that the expression levels of DEGs were significantly different between the ds*HcCht5* and ds*GFP* samples (Figure 4C). To confirm the differential gene expression, ten DEGs were selected for RT-qPCR validation. Most of the selected unigenes exhibited the same expression patterns as those observed in the transcriptome data (Figure S3).

Of all the DEGs, 68% were annotated genes and 32% were unknown (Figure 4B). We found that in addition to *HcCht5*, many other genes related to the chitin metabolism and molting hormone signal were also down-regulated in the ds*HcCht5*-treated group, such as chitin synthase genes (*CHS-A* and *CHS-B*) and β-*N*-acetylglucosaminidase gene (*NAG*)*,* key genes in the chitin synthesis pathway; ecdysteroid kinase gene (*EcK*) and juvenile hormone esterase gene (*JHE*), as well as essential genes encoding molting-related hormones (Figure 4D). These results confirmed the function of *HcCht5* in chitin metabolism and the larval molting process.

Surprisingly, we also found that quite a few down-regulated DEGs were related to detoxification metabolism, including odorant-degrading enzymes (*ODEs*), UDP-glycosyltransferases (*UGTs*), cytochrome P450 (*CYP450*), cytochrome b-561 domain containing protein (*CYB561*), and carboxylesterase (*CaE*) (Figure 4D). Detoxification enzymes have been reported in relation to virous functions in different insect species and are often involved in insecticide, xenobiotic degradation, and pheromone metabolism. In the present study, silencing of *HcCht5* led to the down regulation of detoxification-related genes, suggesting a direct or indirect association of *HcCht5* with detoxification metabolism during molting. To evaluate whether nonspecific dsRNAs have similar effects on immune genes, we examined all the differentially expressed immunity gene expression in untreated insects. The results showed that except for the *ODE1* and *CYB561* genes, the expression levels of all the other immunity genes in the untreated group were consistent with those of the ds*GFP* treatment. This demonstrated that the nonspecific dsRNAs do not have a similar effect on the expression of immunity-related genes (Figure S4).

## 4. Discussion

Insect chitinase genes have been widely recognized as attractive targets for the development of effective and environmentally safe insect management methods [30]. In the present study, we characterized the *H. cunea* chitinase gene (Accession number: U86877) and designated it as *HcCht5*. All insect chitinases belong to the GH18 family and are classified by specific structural domain organization [31]. Based on the sequence analysis, the *HcCht5* gene encoded a 553-amino acid protein with a signal peptide region, a catalytic domain (GH18 domain), and a chitin-binding domain (Figure 1A), possessing four conserved chitinase-catalyzed active sites (Figure 1B). These features were consistent with the characteristics of group I genes in other insect species [1,11]. Phylogenetic analysis also clustered *HcCht5* to the group I chitinase branch (Figure S1). We therefore concluded that *HcCht5* belonged to the group I chitinase branch.

Group I chitinase (*Cht5*) is a well-characterized enzyme found in the integument and molting fluid [1,30]. We detected the expression pattern of *HcCht5* in different tissues of *H. cunea* larvae by RT-qPCR. The results showed that *HcCht5* was highly expressed in the epidermis, similar to the expression pattern of *Cht5* in *P. xylostella* (*PxCht5*) [23] and *Nilaparvata lugens* (*NlCht5*) [32], indicating the important role of *Cht5* in epidermal metabolism. We also examined the temporal expression of *HcCht5* during the different larval stages of *H. cunea*. As shown in Figure 2C, the *HcCht5* transcript was only significantly highly expressed during the molting stage (L4D1 and L4D5), while its expression decreased in the inter-molting stage, which was consistent with the Northern blot results of *HcCht5* (*H. cunea* chitinase) by Kim et al. [25] and similar to the expression pattern of *Cht5s* in *L. migratoria* (*LmCht5-1* and *LmCht5-2*), *P. xylostella* (*PxCht5*), and *C. fumiferana* (*CfChitinase*) [6,14,23], suggesting its important roles in larval molting.

According to previous studies, most research on *Cht5s* in insects focused on molting; however, the function of *Cht5s* in metamorphosis development were slightly different between insects of different orders. In coleopteran, homopteran, and lepidopteran, *Cht5s* have been frequently reported to affect pupal–adult or nymph–adult molting. For example, in *T. castaneum*, a pupa-adult lethal phenotype was observed after the injection of dsRNA for *Cht5*, but the larva-larva and larva-pupa molts were normal [3]; in *P. solenopsis*, silencing *PsCht5* also resulted in pupation defects and failure to complete adult eclosion [24]; in *S. exigua*, injection ds*SeCht5* (ds*SeChi*) at the last instar larvae demonstrated its important role during the larval-pupal and pupal–adult stages [8]. In the orthopteran insect, *L. migratoria*, gene duplication of chitinase 5 (*Cht5-1* and *Cht5-2*) was identified, but only RNAi-mediated suppression of *LmCht5-1* led to severe molting defects and lethality, whereas *LmCht5-2* did not display any visible phenotype [14]. The distinct functions of *Cht5s* in insects may be related to the differences in the developmental patterns and tissue-specificity of chitinase expression. In the present study, the *HcCht5* was demonstrated to be of vital importance in larva-larva molting. The injection of ds*HcCht5* on the fifth day of the third larval stadium resulted in an effective RNAi response accompanied by the knockdown of *HcCht5* expression and arrested molting (Figure 3, Table 1), suggesting that *HcCht5* is absolutely required for the successful molting process of *H. cunea* larvae. Chitinase genes belonging to other chitinase groups have also been linked to different roles in insect development. The function of other *Chts* in *H. cunea* will be assessed in future studies.

Importantly, we found that ds*HcCht5* injection at different larval stages resulted in different RNAi efficiencies. Treatment at the early stage of the fourth instar larvae (L4D1, L4D2 and L4D3) did not cause any phenotypic changes; however, larvae treated at the late days of the fourth instar (L4D4 and L4D5) appeared to experience abnormal molting and mortality (Table 2). In addition, a better RNAi efficiency was detected with injection at L4D5 compared with at L4D4 (Table 2). In other words, the closer to pre-molting, the better the RNAi efficiency was. The RNAi efficiency and the resulting phenotypes were variable in the target insect. These variations are reliant on several critical factors, such as the target gene transcript abundance, spatial and temporal expression profiles, and the protein stability and turnover rate of the target gene [33,34]. In *H. cunea*, we found that *HcCht5* was highly expressed during molting, while its expression decreased by about 40–60 times during inter-molting (Figure 2C), suggesting the function of *HcCht5* in the molting process. When dsRNA injection occurred in L4D1, L4D2, and L4D3, premature intervention may have made it difficult to elicit significant RNAi phenotypes several days later, such as the injection of dsRNA targeting *calreticulin* and *cathespsin-L* in *Acyrthosiphon pisum,* which caused a decrease in target gene expression by 35–41% 1–5 days after injection, stopping at 7 days post injection [35]. Our results demonstrated that RNAi-mediated silencing of *HcCht5* inhibited larval molting depending on dsRNA injection time and provide a reference for the development and application of RNAi-based management of *H. cunea* control.

RNA-seq analysis showed that many genes related to chitin metabolism and molting hormone signaling were also down-regulated after silencing of the *HcCht5* gene. DGE analysis also found that depletion of *HcCht5* decreased the expression of many detoxification enzyme-related genes (Figure 4D). In general, insect detoxification enzymes are often involved in insecticide, xenobiotic degradation, pheromone metabolism, or odorant degradation to maintain the physiological balance within insects [36,37,38,39]. In the present study, genes related to UDP-glycosyltransferases (UGTs) and odorant-degrading enzymes (ODEs) were down-regulated after *HcCht5* RNAi. UGTs catalyze the conjugation of a range of diverse small lipophilic compounds with sugars to produce glycosides, playing an important role in the detoxification of xenobiotics and in the regulation of endobiotics in insects. The expression of insect UGTs has been detected in fat bodies, the midgut, the Malpighian tubules, and even in the antennae, showing different patterns in their expression profiles and suggesting that UGT genes might have different functions [36,40]. Studies have found that many endogenous compounds, such as ecdysteroid hormones and cuticle tanning precursors, are glycosylated by UGT enzymes [36,41]. Recently, a putative UGT from *Heterorhabditis bacteriophora* (*Hb-ugt-1*) was examined, and its activity was found to likely involve the inactivation of ecdysone [42]. ODEs belong to olfactory proteins, playing crucial roles in the responses triggered by external chemical stimuli [43]. Insect ODEs include multiple enzyme families typically expressed in the sensillar lymph and likely involved in the fast inactivation of odorants to maintain the sensitivity of the olfactory system [44]. Few studies have focused on the effect of insect chitinase on detoxification metabolism, although detoxification enzymes are functionally diversified in insects. However, detoxification was considered indispensable for ecdysis. During insect molting, the molting fluid exudes multiple proteins for the recycling of old cuticles, and also produces a great deal of toxic molecules [45]. Successful ecdysis requires all molting proteins to work together, including in detoxification [46,47]. Therefore, we speculated that the down regulation of detoxification-related genes after *HcCht5* RNAi might be due to the inability to molt and the disruption of the insect physiological balance. Further studies are required to confirm this hypothesis.

## 5. Conclusions

Overall, we characterized the *HcCht5* gene in *H. cunea* and detailed its gene function in the larval molting process through RNAi and RNA-seq. Our current findings demonstrated that the RNAi-mediated silencing of *HcCht5* arrested molting and caused larval mortality depending on the dsRNA injection time. The silencing of *HcCht5* down-regulated genes related to chitin metabolism, molting hormone signaling, and detoxification metabolism.

## Figures and Tables

**Table 1:** The abnormal rate and mortality rate of *H. cunea* after injection of ds*HcCht5* in different concentrations.

Treatment ^1^	ds*GFP*	ds*HcCht5*	ds*GFP*	ds*HcCht5*
	2 ug/Larva		4 ug/Larva	
Abnormal rate	ND	33.3%	ND	66.7%
Mortality rate	ND	33.3%	ND	66.7%

^1^ A total of 30 insects on the fifth day of the third instar were injected for each treatment group. The abnormal rate was investigated at molting stage of the third instar larvae. The mortality rate was investigated five days after treatment. ds*GFP*, the control group; ds*HcCht5*, the treatment group; and ND, not detected.

**Table 2:** The abnormal rate and mortality rate of *H. cunea* after the injection of ds*HcCht5* at each day of the fourth larval stadium (L4D1 to L4D5).

Treatment ^1^		ds*GFP*	ds*HcCht5*
Abnormal rate	L4D1	ND	ND
	L4D2	ND	ND
	L4D3	ND	ND
	L4D4	ND	10.0%
	L4D5	ND	26.7%
Mortality rate	L4D1	ND	ND
	L4D2	ND	ND
	L4D3	ND	ND
	L4D4	ND	10.0%
	L4D5	ND	26.7%

^1^ A total of 30 insects were injected with 4 ug ds*GFP*/ds*HcCht5* at each day of the fourth larval stadium (L4D1 to L4D5). The abnormal rate was investigated at the molting stage of the fourth larval stadium. The mortality rate was investigated five days post molting. ds*GFP*, the control group; ds*HcCht5*, the treatment group; and ND, not detected.

## Data Availability

The data presented in this study are available in article.

## Supplementary Materials

The following are available online at https://www.mdpi.com/article/10.3390/insects12050406/s1, Table S1: Primer sequences used in this study, Table S2: GenBank accession numbers for the phylogenetic tree used in this study, Table S3: Expression stability values of the reference genes for different developmental stages and different tissues, Table S4: Summary of the RNA sequencing data, Table S5: DEGs between the ds*GFP* and ds*HcCht5* samples, Figure S1: Phylogenetic tree of chitinases from different insect species, Figure S2: Correlation analysis of RNA-seq samples, Figure S3: RT-qPCR validation of 10 selected RNA-seq-based DGEs, Figure S4: The expression levels of the differentially expressed immunity genes in the untreated group, ds*GFP*-treated group, and ds*HcCht5*-treated group.
