# Supplementary material for: RNAi-Mediated Silencing of the Chitinase 5 Gene for Fall Webworm (Hyphantria cunea) Can Inhibit Larval Molting Depending on the Timing of dsRNA Injection
Source: Insects. 2021 Apr 30;12(5):406. doi: 10.3390/insects12050406 (PMC8147239; doi:10.3390/insects12050406)

Table S1. Primer sequences used in this study

Primers used for HcCht5 coloning

Primer name	Primer sequence (5'-3')
HcCht5-F	ATGCGAGTCTTACTAGCGTCGTT
HcCht5-R	CTATTTCCTGCAGTTATTGCGAT

Primers used for dsRNA synthesis

Primer name	Primer sequence (5'-3')
dsGFP--T7F	taatacgactcactatagggTGAGCAAGGGCGAGGAG
dsGFP--T7R	taatacgactcactatagggCGGCGGTCACGAACTCCAG
dsHcCht5-T7F	taatacgactcactatagggCACGCATCTCATCTACTCA
dsHcCht5-T7R	taatacgactcactatagggCGAACCTTTACCGACCCT

Primers used for RT-qPCR detection of HcCht5

Primer name	Primer sequence (5'-3')
HcActin-qF	GGTTACTCTTTCACCACCACAG
HcActin-qR	GGACTTCTCAAGGGAACTGC
HcCht5-qF	TCGGTCGTTCACTTTAGCAG
HcCht5-qR	TTTGTAAGCGTAGGGGCAT

Primers used for RT-qPCR validation of selected DEGs

Primer name	Primer sequence (5'-3')	Gene ID
BG1-qF	AGCAATGACTTCAAGCCTACA	c46905.graph_c0
BG1-qR	AACCCTGTCTCCTGACTTCG	
JHE-qF	CGGTTTAACAACATTTGTCCC	c40455.graph_c0
JHE-qR	AAGGTTTCGCACTCAGCATT	
EcK-qF	AGTAGCTGATTCGATCTTGGTC	c39726.graph_c0
EcK-qR	TTTCCTTGTTTCGTAGTCCGT	
CPA2-qF	GGAACATCGGGTCTCGTCTT	c47956.graph_c0
CPA2-qR	GCCTGGTTACTCTGGGGAAT	
ODE1-qF	CGTCAGCCAGACCCAAGATG	c43328.graph_c1
ODE1-qR	GCCCGACAGGTAGAAGGACA	

ODE2-qF	GCAGCTCTTAGATGGGTGAA	c47555.graph_c3
ODE2-qR	TCTGGGTAATGGCTCTTTTG	
UGT1-qF	ATCCAGCTTTGTCACCACATG	c45767.graph_c0
UGT1-qR	CGGTCCCCTTGGAACTTTTC	
UGT2-qF	CAATCAACAGACGAAGCTATCAC	c47118.graph_c0
UGT2-qR	CTAAATCAAGCCGGACTCCAA	
CYP450-qF	TAATAACGAGCCAGGAAACAG	c47822.graph_c2
CYP450-qR	TTCGTCAAGTAGTGGGGTGTA	
CaE-qF	AAATATGGAAACCCAACCCC	c47555.graph_c2
CaE-qR	GATGGCATCCCAAAACTTGAT	

Table S2. GenBank accession numbers for the phylogenetic tree used in this study.

Species	Gene name	GenBank accession no.
Acromyrmex echinatior	AeCHT3	EGI59292
Ancylostoma ceylanicum	AcCHT7	EYC03522
Anopheles gambiae	AgCht1	XP_317335.2
	AgCht2	XP_315650.4
	AgCht4	XP_315351.4
	AgCht7	XP_308858.4
	AgCht10	XP_001238192.2
	AgCht11	XP_310662.5
	AgCht13	XP_314312.4
	AgCht16	XP_319801.4
	AgCht23	XP_001688641.1
	AgCht24	XP_316256.4
	AgIDGF2	XP_001237925.1
	AgIDGF4	XP_317398.3
	AgCHT5-1	HQ456129
	AgCHT5-2	HQ456130
	AgCHT5-3	HQ456131
	AgCHT5-4	HQ456132
	AgCHT5-5	HQ456133
Bombyx mori	*BmCht1*	XP_004931749.1
	BmCht2	XP_004933352.1
	BmCht3	XP_012551241.1
	BmCht5	AAB47538.1
	BmCht6	XP_012553393.1
	BmCht7	XP_004922005.1
	BmCht11	XP_004926923.1
	BmCht-h	BAC67246.1
Daphnia pulex	DpCHT5	DpCHT5
	DpCHT6	DpCHT6
Drosophila melanogaster	DmCht1	NP_609190.2
	DmCht2	NP_477298.2
	DmCht5	NP_650314.1
	DmCht6	NP_572598.3
	DmCht7	NP_647768.3
	DmCht10	EAA46011.1

	DmCht11	NP_572361.1
Locusta migratoria	LmCht10	AMT75074.1
Ostrinia furnacalis	OfCht-h	BAE16587.1
Tribolium castaneum	TcCht1	XP_971647.1
	TcCht2	XP_970191.2
	TcCht3	XP_008197064.1
	TcCht5	NP_001034524.1
	TcCht7	NP_001036035.1
	TcCht10	NP_001036067.1
	TcIDGF4	NP_001038091
	TcIDGF2	NP_001038092.1

Table S3. Expression stability values of the reference genes for different developmental stages and different tissues.

Different developmental stages			Different tissues		
Gene	Stability value	Rank	Gene	Stability value	Rank
β-actin	0.597	1	*β-actin*	0.720	1
β-tubulin	0.651	2	*β-tubulin*	0.733	2
GAPDH	0.750	3	*GAPDH*	1.274	3
EF1α	1.160	4	*EF1α*	1.345	4

Table S4. Summary of the RNA-sequencing data..

**Samples**	**Clean reads**	**Clean bases**	**Q20 (%)**	**Q30 (%)**	**GC (%)**
ds*HcCht5*-1	26,222,045	7.8G	97.73	94.51	45.71
ds*HcCht5*-2	28,097,827	8.4G	97.73	94.52	45.47
ds*HcCht5*-3	25,301,548	7.6G	97.71	94.49	45.36
ds*GFP*-1	26,224,702	7.8G	97.72	94.50	45.38
ds*GFP*-2	24,600,974	7.4G	97.60	94.27	46.38
ds*GFP*-3	24,480,876	7.3G	97.70	94.45	45.81

Figure S1. Phylogenetic tree of chitinases from different insect species. The tree was generated using MEGA 6.0 software with neighbor joining method. Bootstrap analyses of 1000 replicates were performed, and the bootstrap values are represented in cladograms. Chts are grouped into 11 different groups. Cht of H. cunea (HcCht5) is marked with a red square. The GenBank accession numbers of these chitinases are listed in Table S2.

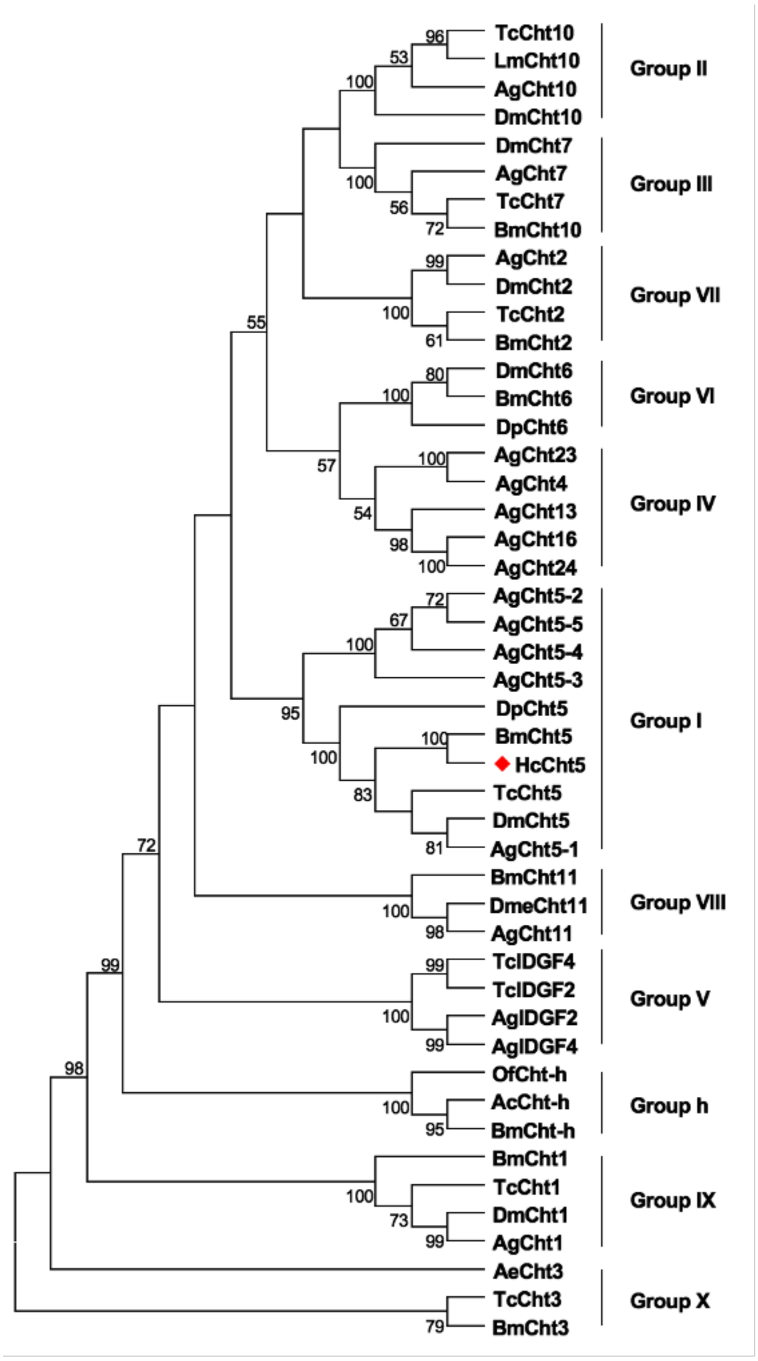

Figure S2. Correlation analysis of RNA-seq samples. Pearson's correlation coefficient was used as the evaluation index of inter-sample correlations. $r^2>0.8$ represents a strong correlation of all the samples.

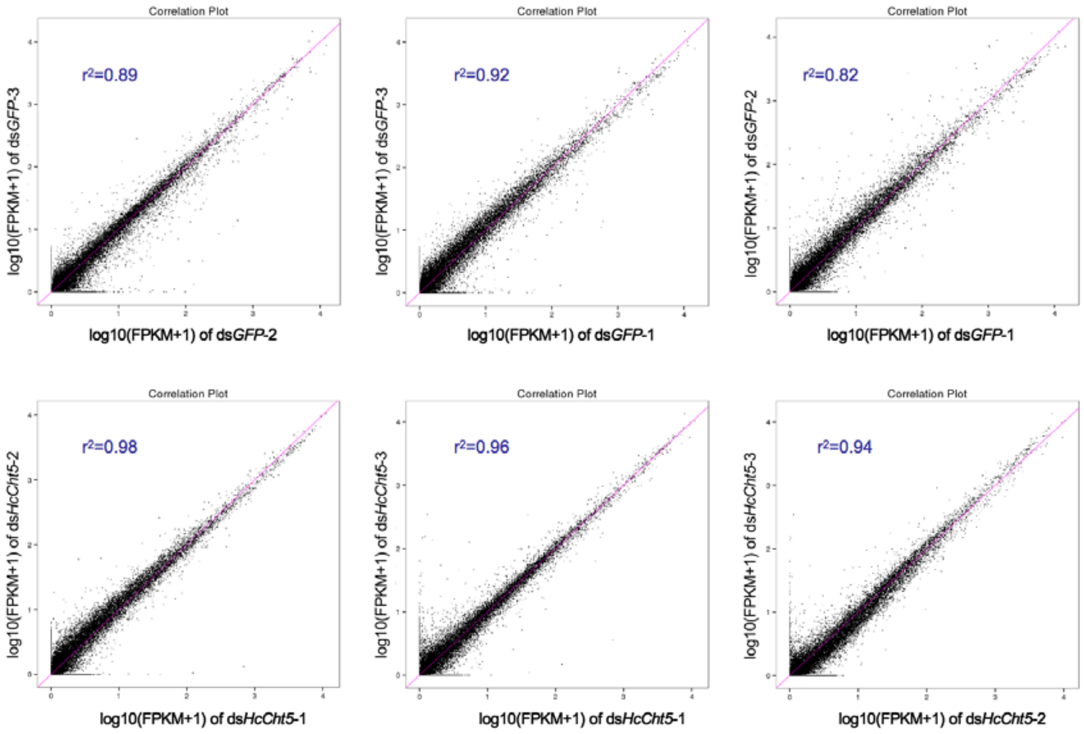

Figure S3. RT-qPCR validation of 10 selected RNA-seq-based DGEs. The letters indicate putative gene names in *H. cunea*.

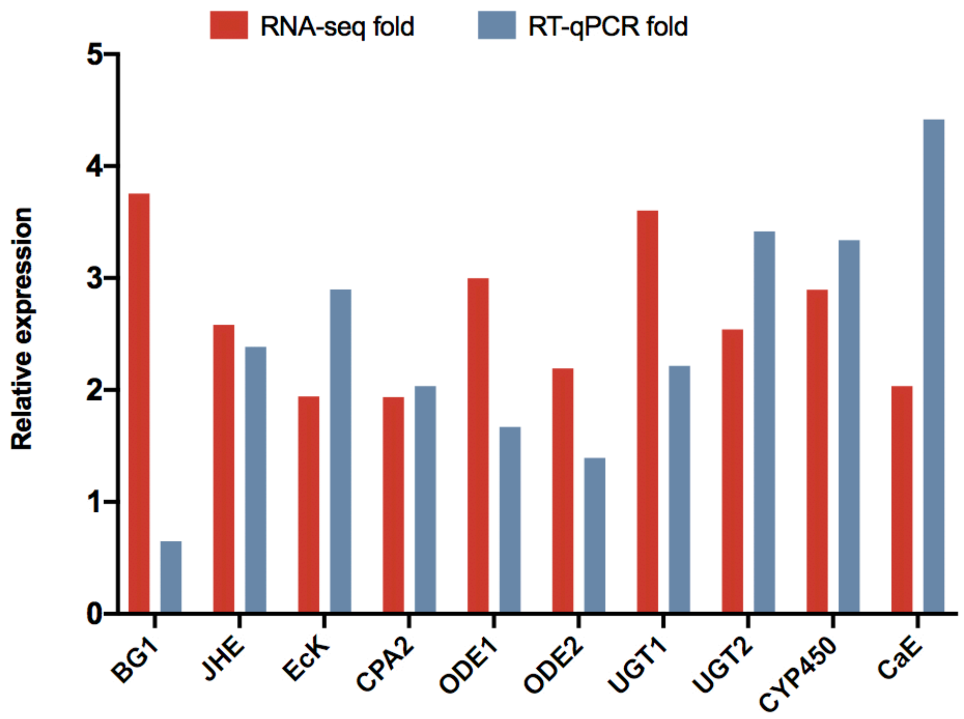

Figure S4. The expression levels of the differentially expressed immunity genes in the untreated group, ds*GFP*-treated group, and ds*HcCht5*-treated group (*, P < 0.05; and **, P < 0.01).

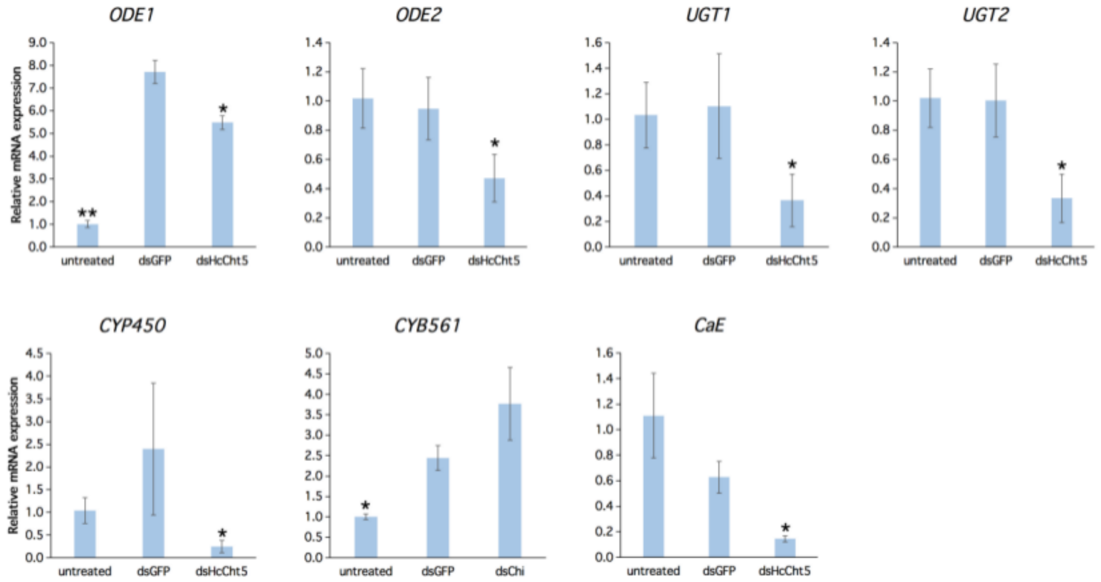

Supplement: Supplementary file 1 [file insects-12-00406-s001.zip › sup. Table S1-S4; Fig. S1- S4.pdf]
